# Turn-taking: a case study of early gesture and word use in answering WHERE and WHICH questions

**DOI:** 10.3389/fpsyg.2015.00890

**Published:** 2015-07-08

**Authors:** Eve V. Clark, Kate L. Lindsey

**Affiliations:** Department of Linguistics, Stanford University, StanfordCA, USA

**Keywords:** where and which questions, answers, gestures, words, timing

## Abstract

When young children answer questions, they do so more slowly than adults and appear to have difficulty finding the appropriate words. Because children leave gaps before they respond, it is possible that they could answer faster with gestures than with words. In this study, we compare gestural and verbal responses from one child between the ages of 1;4 and 3;5, to adult **Where** and **Which** questions, which can be answered with gestures and/or words. After extracting all adult **Where** and **Which** questions and child answers from longitudinal videotaped sessions, we examined the timing from the end of each question to the start of the response, and compared the timing for gestures and words. Child responses could take the form of a gesture or word(s); the latter could be words repeated from the adult question or new words retrieved by the child. Or responses could be complex: a gesture + word repeat, gesture + new word, or word repeat + new word. Gestures were the fastest overall, followed successively by word-repeats, then new-word responses. This ordering, with gestures ahead of words, suggests that the child knows what to answer but needs more time to retrieve any relevant words. In short, word retrieval and articulation appear to be bottlenecks in the timing of responses: both add to the planning required in answering a question.

## Introduction

When adults answer questions, their answers are surprisingly fast, regardless of the language involved. The median gap between the end of a yes/no question, for example, and the start of the answer, is about 200 ms for speakers of English, with a range over other languages from 0 ms to nearly 400 ms (Stivers et al., 2009). This close timing entails that the addressee begins planning an answer before the speaker finishes the question. This timing reflects the general principle of ‘no gap, no overlap’ as speakers participate in conversation.

Young children take much longer to answer questions than adults do (Casillas, 2014a), apparently waiting until they have heard the full question before they start to plan an answer. To interpret a question, children must first process its content. [Between 1;6 (1 year, 6 months) and 3;0, they get much faster, almost to adult speed, in recognizing familiar words (e.g., Zangl et al., 2005; Fernald et al., 2006)]. Once they have done this, they can then decide on and formulate an answer. But they may take up to 2 s, or more, before they start their answer after a question. The size of this gap between question and answer decreases over time for specific question-types (Casillas et al., under review), and by around 3;6–4;0, children often manage near-adult timing.

To date, studies of how children answer questions have focussed on verbal answers, where children either repeat one or more words from the adult question in their answer, or construct a verbal answer from scratch. This suggests that one factor in answering a question is how much word production children have to plan in order to come up with an answer. When they repeat a word from the adult’s question as the answer, they need not retrieve anything from memory, so the cost should be minimal. But when they have to retrieve one or more appropriate words, and the relevant construction, from memory before they begin to articulate their answer, the cost should be much greater.

What happens, though, when children (or adults, come to that), have the option of answering the question with a gesture, either alone or in combination with speech? Once children have decided on an answer, to extend hand and arm to designate a place in answer to a *Where* question, or to select an alternative in answer to a *Which* question, would seem to require less planning and articulation than producing an utterance, where children must first decide which words to repeat of those already said, say, or else search for and retrieve one or more appropriate words and then articulate those in their response.

### Gestures in Young Children

Children start to produce manual gestures fairly early to indicate interest in or desire for something in their immediate environment. They start to point (interest: ‘look’) and reach (desire: ‘I want’) from as early as 10–12 months. These two gesture-types have been considered to be proto-speech acts for asserting and requesting (Werner and Kaplan, 1963; Wundt, 1973; Bates et al., 1975; Bruner, 1975). The vast majority of young children’s early gestures appear to be deictic in nature, mainly index-finger POINTS, but they also produce gestures to SHOW, OFFER, or PLACE objects for the other (see Caselli, 1990; Liszkowski, 2006; Andrén, 2010). In his case study of five children up to age 2;6, Andrén (2010) found that these gestures were nearly always (94%) coordinated with speech, with only a few gestures overall occurring on their own (see also Kelly, 2014).

Children demonstrate comprehension as well as production of POINTS from around 12 months on (e.g., Muñetón Ayala and Rodrigo López, 2011; Behne et al., 2012). When adults look and point, children treat this as a directive to look at whatever is being targeted. In general, adult gaze and pointing are critical in establishing joint attention (e.g., Carpenter et al., 1998; Estigarribia and Clark, 2007).

By 1;6, children appear to use POINTS differentially: to elicit a term for something, they simply point and look, and adults then typically offer a label for that referent. But when children combine a point with vocalization or a word, adults are significantly more likely to treat that combination as a request and to give the child the object targeted (see Carter, 1978; Kelly, 2011; Olson and Masur, 2011).

### *Where* and *Which* Questions

When young children answer questions, they may substitute an indicating gesture for speech, for example answering a *where* question with a POINT to the relevant location. In this study, we compare gestural and verbal responses to adult *Where* and *Which* questions addressed to one child between the ages 1;4 and 3;5.

To make this comparison, we examined the relative timing of any gesture onset in an answer, compared to the timing of any vocal or verbal onset in the answer. Answers could take several forms: (a) they could be simple, containing just a single response-element: a gesture, a babble (vocalization), or a word—either a word repeated from the adult question or a new verbal choice made by the child; or (b) they could be complex, consisting of two elements: a gesture + babble, gesture + repeat, gesture + new-word(s), or repeat + new-word(s).

Since gestural responses do not require any lexical retrieval, we expect that children should be able to produce a gestural response as soon as they have understood the question. Producing a new verbal response should be more complex and therefore take more time: the child must retrieve any relevant word(s), plan the answer, and then articulate it. So any measurable difference between gestural and verbal response times would provide a preliminary indication of question-comprehension time (how long it takes to issue a gesture) and hence how much time is required to produce a verbal response.

If the child produced a gesture in a combined gesture + verbal response, with the gesture produced ahead of the child’s word(s), this would be evidence that the child knows what to answer but needs additional time to retrieve relevant words. The conceptual cost of identifying a relevant answer is the same for gestural and verbal responses. But for verbal responses, lexical retrieval is one bottleneck in the timing of an answer, followed by planning the response and articulation (Levelt, 1989; Levinson, 2000, 2006). We would therefore expect that repeats of one or more words from the adult question, available in short-term memory, should take less time than when the child constructs an answer with words newly retrieved from memory. In short, both lexical retrieval and articulation are added costs and so should take longer, as shown schematically in **Table 1**. In the present case study we focus on the processing costs in the production of verbal responses compared to gestural ones.

**Table 1:** Costs (effort) required in answering a question.

	Lexical retrieval	Articulation
Gesture	–	–
Babble	–	(√)
Repeated word(s)	–	√
New word(s)	√	√

## Materials and Methods

For this study, we drew on the corpus for one child, Alex, from the Providence Corpus of American English (Demuth et al., 2006), in the Child Language Data Exchange System archive (CHILDES; MacWhinney, 2000). All data in the CHILDES Archive were collected in accordance with the internal review board on human subjects of the relevant university, with permission for use of the data in further analyses by researchers not involved in the original study. This corpus contains high-quality video recordings of biweekly spontaneous interactions, each lasting 1–1.5 h, between parent and child. Each video session contains numerous parental questions and child answers. This allowed us to measure both gestural and verbal onset times in the child’s responses to parental questions. Recordings for Alex were made approximately every 2 weeks, beginning at age 1;4.20 until 3;5.15, for a total of 51 video sessions. For our analysis, we chose 26 of these sessions, one per month, to capture snapshots of Alex’s development. If any particular month had fewer than 10 tokens of the relevant question–answer types, and if there was an additional video available in that month, we drew on both video sessions. We did this for nine sessions—at 1;7, 1;11, 2;2, 2;3, 2;8, 2;10, 3;1, 3;2, and 3;4—so each of these months was represented by two data sessions from Alex, for a total of 35 videos in all.

In order to analyze the time it took for the child to answer his mother’s questions, we compared gestural and verbal responses. Gestural responses offer a non-verbal form of response that avoids the need to find appropriate words, while verbal responses require finding and producing an appropriate answer. *Where* and *Which* questions can be answered with either a gestural or verbal response, or both. We extracted all *Where* and *Which* questions, and measured the onset times of all responses provided by the child. We identified a total of 502 *Where* and *Which* questions in the 35 videos selected, and then identified all the responses given. The adult (mother) in each case treated the child’s responses as answers to the question just asked. Moreover, the majority of these responses explicitly provided relevant semantic information: a target-place in response to *Where* questions, and a chosen alternative in response to *Which* questions. The one response-type we were unable to assess was vocal babble on its own: these babbles were consistent in form but, to us, uninterpretable. His mother, however, treated these too as appropriate responses.

### Coding

We coded all the child responses using the following categories: (i) *gesture*: manual gesture (G) to the target object’s location, (ii) *speech*: babble (B), repeat of a term from the adult question (R), new verbal response (V), and (iii) *location*: on camera or off camera (O). We also coded as ‘no response’ (N) any questions the child failed to answer, questions where the child was impeded from responding (i.e., while eating, or with a body position that delayed a possible gestural response), and questions asked when the child was already manipulating, or speaking about, the relevant object.

We collected metadata for each question/response pair, including: (i) age of child, (ii) video file, (iii) timestamp for that question in the video session (HH:MM:SS), (iv) question type (*Where* or *Which*), (v) the adult’s actual question, and (vi) the content of the child’s response. Next, we used a Python script to extract 12 s of audio/video for each of the 502 questions we had identified, beginning 2 s before each question onset. Each video clip was imported into ELAN and paired with a synchronized transcription file. The onsets and endings of all questions, and of all answers (gestural and verbal) were transcribed into question, gestural, and verbal tiers, respectively. Adult *Where* and *Which* questions received 235 (47%) unimpeded gestural and/or verbal responses from the child.

We then measured, in milliseconds, the time from the ending of each question to the onset of the answer. Question endings were marked following complete expulsion of sound, including aspiration. Verbal onsets were marked immediately prior to any vocal utterance (including utterance-initial “um”). Gesture onsets were marked immediately prior to the beginning of movement (the preparation of the gesture), and duration of the stroke was also measured where possible. In the nine instances where a question was repeated multiple times, we used our discretion in determining which instance the child was attending to. Lastly, we excluded outlier responses that were two SDs or more from the relevant mean (13 answers, 5.5%) from our statistical analyses.

The timing of 10% of Alex’s responses to *Where* and *Which* questions was rechecked by an independent coder. This coder and the second author agreed on the timing measurements, within 200 ms (the smallest discriminable difference) 88% of the time, with high inter-rater reliability (Pearson’s *r* = 0.930, *p* < 0.0001). When the timing window was expanded to within 400 ms, the agreement rate rose only very slightly, to 91%.

## Results

Of these 221 questions included in our statistical analyses, *Where* questions received 137 responses, and *Which* questions 84 responses. ‘Single’, simple, responses followed 145 (66%), and ‘double’, complex, responses followed 76 (34%). In the double responses, we measured the onset timing for each element separately (76 × 2). The single or simple responses consisted of one element in the response. There were 14 gestures, 13 babbles, 56 repeats, and 62 new-word responses (i.e., words retrieved specifically for the response given), as shown in **Table 3**. Double or complex responses consisted of those where the child combined a gesture with a vocalization or word, or combined a repeat from the adult’s question with some added verbal material. These combinations were ordered, with 10 gesture + babble responses, 7 gesture + repeated word, 39 gesture + new-word, and 2 repeated word + new-word responses, along with another 18 other complex answers where the second element seemed to be produced for added clarification rather than being an integral part of the initial response. In these cases, Alex produced an indicating gesture *after* saying a repeated or new word. In seven instances, he babbled and then, after a pause, pointed; in five more, he repeated a word from the question, and only then pointed; and in another five, he produced a new word and then pointed. In each instance, Alex appeared to be trying to clarify his answer by adding the gesture afterward (see **Table 4**, right-hand columns). And in one, he produced a one-word answer, paused, and then repeated a word from the adult question. We therefore excluded the timing for these from our overall computations of the relative timing of the two parts in double responses.

In all, we measured 297 response tokens (145 tokens from the simple responses, and 152 tokens from the 76 complex, double responses). The overall mean response times, from the end of the question to the onset of the child’s answer, are summarized in **Table 2**.

**Table 2:** Mean response times (in milliseconds) for all response-types.

Response-type	*N*	Time to response onset
Gesture	70	504
Babble	23	725
Repeat	65	717
New word	103	957

Overall, Alex’s gestures were produced faster than his babble responses. His babbles, repeats of adult words, and new-word responses differed significantly overall [*F*(3,259) = 9.355, *p* < 0.0001], with babbles and repeats both produced significantly faster than new-word responses (**Table 2**). His gestures were faster than both repeated word responses (Fisher’s Least Significant Difference test, *p* < 0.021), and new-word responses (Fisher’s LSD, *p* < 0.0001). And his repeated word responses in turn were significantly faster than his new-word responses (Fisher’s LSD, *p* < 0.009). Finally, within his complex double responses (**Table 4**), his gestures were significantly faster than the verbal responses they were paired with, where the gesture onset preceded the word onset [*t*(56) = 7.408, *p* < 0.0001].

Although we combined the responses to *Where* and *Which* questions in the overall analysis, inspection of the two questions and their responses separately showed the same patterns in onset timing for the response types: Gestures (*Where* 598 ms, *Which* 379 ms) were produced faster than any verbal responses. Among verbal responses, babbles and repeats (*Where* 1056, 753; *Which* 580, 353) were faster than new-word responses (*Where* 1103; *Which* 848), and repeats in turn were faster than new-word responses.

When we compare these response-types in single responses (**Table 3**) to double responses (**Table 4**), we see that the mean onset timing for gestures and verbal responses varies with the complexity of the answer. It also varies for verbal responses, whether the child repeats one or more of the words he has just heard, combines one or more repeated words with a word newly retrieved from memory, or constructs a verbal response of one to three or more words that are entirely new. Although responses to some question-types get faster with age (Casillas et al., under review), such gains are often obscured by the fact that, as children get older, they also start to produce some longer, more complex answers where earlier they had produced only one word.

**Table 3:** Mean response times (in milliseconds) for single response-types.

Response-type	N	Time to response onset
Gesture	14	788
Babble	13	685
Repeat	56	678
New-word	62	950

**Table 4:** Mean response times (in milliseconds) for double response-types, with the basic order of the two elements produced.

Response-type	*N*	Time-1	Time-2	Response-type	*N*	Time-1	Time-2
Gesture + Babble	10	258	778	Babble + Gesture	5	553	678
Gesture + Repeat	7	567	916	Repeat + Gesture	7	805	984
Gesture + New-word	39	454	919	New-word + Gesture	5	784	1422
Repeat + New-word	2	1101	1919	New-word + Repeat	1	546	1209

The single gesture responses consistently indicated the location in response to *Where*, and the alternative-chosen in response to *Which*. Babbles, produced on their own and in some double responses, may have had some attention-getting function, especially when combined with a pointing gesture. On their own, they appear to have been interpretable to the mother as early attempts at words: she treated them all as responses. The verbal elements in single responses consisted either of one or more words repeated from the adult question (*n* = 56)—typically the word for the object being sought (*Where?*) or for the object chosen (*Which?*)—or of semantically relevant unmentioned words retrieved by the child from memory (*n* = 62). Initially, Alex tended to repeat words, mainly single words, from the adult question (e.g., *papa, balloon*, etc.), but as he got older, he also began to produce deictic terms like *there* or *that*, either on their own or in combination with repeated or newly retrieved words. His verbal responses also became longer with age, and by 2;4–2;6, he produced some answers of three words or more [e.g., Which shoes, the green ones? —*The green ones* (2;6); Where would you like to sit, right here? —*Over there* (2;8); Where’s the baby going? —*Baby go in a stroller to go for a walk* (3;0); Which one would you like to paint? —*I would like to paint this one* (3;1)].

Does planning a longer verbal response take longer? We looked at the correlations with age for Alex’s responses (a) where he repeated one vs. two vs. three or more words from the adult question, and (b) where he produced one vs. two vs. three or more words retrieved from memory. In the first case, where he had just heard the word(s) he repeated in the adult’s question, there was no change with age, whether he repeated 1, 2, or 3+ words (*r* = 0.085, n.s.), but when Alex constructed his answer with his own words, he produced a larger number of more complex (longer) utterances as he got older (*r* = 0.225, *p* < 0.043). We also looked, within his single responses, at how long he took to produce 1–, 2–, and 3+ word answers. On average, longer answers took longer to produce for both repeats and new-word responses, as shown in **Table 5**. However, there was effectively no correlation between length-of-response and timing here, largely because both repeats (*r* = 0.108, n.s.) and new words (*r* = 0.149, *p* < 0.065, n.s.) displayed wide variance in timing with production of utterances of the same length. This is attributable to extraneous factors such as how well the child was actually attending when the adult issued the question, whether he could remember immediately where something was, and his basis for deciding which alternative to choose.

**Table 5:** Alex’s response length in words and timing in milliseconds.

Repeats			New words		
Length	*N*	msecs	Length	*N*	msecs
1 word	41	669	1 word	38	867
2 words	16	752	2 words	39	877
3+ words	6	818	3+ words	28	1109

As children get older, they get better at planning and better at articulating words and sequences of words, so they can produce longer answers. But they should also gradually speed up, perhaps doing so more readily when they repeat words from the adult question than when they construct an entirely new answer themselves. This would initially yield the different means for responses where Alex repeated one or more words from the adult question, compared to where he constructed a new answer with words retrieved from memory (see **Table 5**). As Casillas et al. (under review) noted, young children do get faster at answering *yes/no* questions as they get older. However, children’s increasing speed in answering *Wh-*questions is often obscured by their ongoing acquisition of different *Wh*-words, and the added planning needed to answer *Wh-*questions, as children master the meaning of each *Wh*–type in turn. Of *Where* and *Which, Where* is typically acquired first (Ervin-Tripp, 1970; Tyack and Ingram, 1977), and Alex answered *Where* questions almost twice as often as *Which* (137 to 84). However, we did not have enough data to detect any changes in speed for Alex’s answers to these *Wh*- questions over time, as he got older.

## Discussion

We analyzed the response times for all answer-types and, as expected, found that Alex’s gestures were produced the fastest overall. This finding is consistent with our expectation that both word-retrieval and articulation add costs to responding. Young children struggle to retrieve words from memory, and also have a hard time producing words in recognizable form. When we limited our analysis to his verbal responses, we found that Alex took less time overall when he relied on one or more words repeated from the adult’s question than verbal responses when he retrieved and produced specific words of his own. Here too, we see a cost for word-retrieval, as against simply repeating words available in short term memory. In double answers, Alex generally produced gestures ahead of words, suggesting that he typically knew what he wanted to answer, but needed time both to retrieve words and to articulate them. In short, word retrieval is one bottleneck in the timing of responses: it adds to the planning cost required in answering a question.

### Recognizing and Retrieving Words

Children get better at recognizing familiar words as they get older. They speed up steadily from 15 to 24 months, at which time they come close to achieving adult speed in recognizing familiar words (see Fernald et al., 2006, 2013; Fernald and Marchman, 2012). They also steadily improve in recognizing partial words, and in processing words that have been mispronounced (e.g., Swingley and Aslin, 2000; Swingley, 2009).

Recognizing familiar words, though, is a rather different matter from retrieving those words and producing them in order to answer a question. During their second year, as children’s production vocabularies begin to expand, they make numerous errors in production, often retrieving the wrong word. Dapretto and Bjork (2000) found that children between 14 and 24 months with larger vocabularies were more likely to be able to retrieve the appropriate words for objects that had been hidden in a box, and that with pictures as prompts they could generally retrieve the appropriate words. They also found that retrieval errors were very frequent in naturalistic picture-book reading, for those children whose production vocabulary had just begun to increase, compared to those who still had only a very small vocabulary or those with a relatively large vocabulary toward the end of the second year. Retrieving the right words early on, then, gives young children much more difficulty than recognizing words they hear from others. This is consistent with the general advance of comprehension over production (see Clark and Hecht, 1983).

But in order to answer questions, children need to be able to retrieve the right words. This in turn depends on their having already made the appropriate mapping as they linked forms and (preliminary) word meanings so they could recognize those words from others—for comprehension. This first step is essential for children trying to retrieve the relevant or most appropriate word(s) for production. Question-answering depends on both comprehension and production, with comprehension of the question followed up by (a) the idea of a possible answer, and (b) its instantiation as a gesture, as an utterance, or as some combination of the two (see **Tables 3** and **4**). But responding with an utterance requires that children be able to retrieve any pertinent words and, if necessary, combine two or more words in a syntactic construction, for subsequent articulation.

### What Role does Articulation Play?

In learning how to produce a word, children need to produce it in a form that is recognizable to the addressee, but that may take quite a long time to achieve. Children’s early attempts at words often fail because they do not produce a recognizable word and because they produce different variants each time they try to say that word (see, e.g., Dromi, 1987). This, of course, makes it harder for adults to recognize what the child is trying to say. At the same time, if children produce *consistent* word forms, as when children rely on idiosyncratic templates (e.g., /babiŋk/ for ‘blanket’), even if these fail to match the adult targets, adults can generally understand what the children intend. But children continually monitor and fine-tune their production, eventually matching the conventional forms produced in the speech community around them. One way to characterize their articulatory development is in the form of two ‘rules’ for early word production, *Be consistent* and *Be precise* (McAllister Byun et al., 2016). The former allows for recognition by others, across different contexts, of words articulated in a non-standard way, and the latter captures the fact that children try hard to emulate the conventional forms they hear from adults. Learning how to articulate single words and then sequences of two or more words takes effort, and acquiring the adult pronunciations takes time (Stoel-Gammon, 2011). In short, articulating any kind of verbal response to a question takes time, over and above the time needed to retrieve words from memory. Planning a response, from deciding on an appropriate conceptual answer, to finding the words and organizing them into an appropriate construction, to articulating the relevant utterance, all takes time (Levelt, 1989; see also Levinson, 2006).

Finally, in relying on gestures alone and on gestures along with some speech, children are following adult usage. When adults take turns, and, for example, ask and answer questions, they can do this using speech or gesture, or both (Clark, 2012). And gestures are often used in interaction to identify the place of some target referent, along with a description, or in lieu of a description (e.g., Bangerter and Oppenheimer, 2006; de Ruiter et al., 2012). In short, gestures alone can serve as turns; they can also be combined with speech. We have made use of this usage in young children in order to unpack some of the steps involved in identifying and then planning a verbal answer.

### From Answering Questions to Turn-Taking and Interaction

Turn taking is fundamental in human interaction. Even in infancy, babies respond to parental talk and gaze, initially with extensive overlapping with adult speech in their vocalizations (e.g., Ginsburg and Kilbourne, 1988; Van Egeren et al., 2001). As infants get older, they produce fewer overlaps and more turn-like interactions using gaze, babble, and early word forms (e.g., Rutter and Durkin, 1987; D’Odorici et al., 1997; Hilbrink et al., 2014). But the gaps they leave between turns are often too long. Once they start to use words in their own turns, young children become more adept at anticipating who will talk next when watching an ongoing conversation and they look to the next speaker at appropriate turn-switch points (see Casillas and Frank, under review). But being able to track what is happening in a conversation between third parties is just part of managing interaction. Children also have to learn to take turns on time for themselves.

When people ask questions, they expect to hear answers in the next turn, and the median gap between question and answer for *yes/no* questions in English for adults is around 200 ms (Stivers et al., 2009). But at age one and two, children take considerably longer in producing their answers, although they slowly speed up over the next few years (Casillas et al., under review). And although they get faster in answering simple *yes/no* questions, tracking their increase in speed with age is complicated by the fact that they are simultaneously adding new *Wh*-question words (e.g., *who, how, when*, etc.) to their repertoire, and learning how to answer each question type. Yet they clearly know, by age two if not earlier, that they are expected to answer as soon as they can. At this point, they begin to produce floor-holders like *um*, or start an utterance and keep repeating the first word (e.g., *That– that– that*–…) until they have their full answer ready (Casillas, 2014b).

Turn-taking is a skill fundamental to language use: it is critical for coordinating with others, whether to exchange greetings, answer questions, exchange information, collaborate in all sorts of activities, or co-construct a story. While some form of turn-taking, with attention to gaze, for example, first emerges during infancy, it is only once children produce recognizable words that they begin to participate in conversational exchanges, and begin trying to observe the adult’s ‘No gap, no overlap’ pattern of taking turns.

Indeed, turn-taking is central to all conversational interaction: it relies on gaze and gesture as well as on the child’s utterances. Examining different aspects of turn-taking and, in particular, how children answer different question-types, allows us to take a closer look at how children make use of what they know about both language and interaction so far (Arnon et al., 2014; Grüter and Paradis, 2014). They not only learn language in interaction, but, in interacting, display what they already know and how readily they can process it for production.

## Conflict of Interest Statement

The authors declare that the research was conducted in the absence of any commercial or financial relationships that could be construed as a potential conflict of interest.
